# Use of Chitosan-Based Polyelectrolyte Complexes for Its Potential Application in Active Food Packaging: A Review of Recent Literature

**DOI:** 10.3390/ijms241411535

**Published:** 2023-07-16

**Authors:** Nancy Alvarado, Romina L. Abarca, Cristian Linares-Flores

**Affiliations:** 1 Grupo QBAB, Instituto de Ciencias Aplicadas, Facultad de Ingeniería, Universidad Autónoma de Chile, El Llano Subercaseaux 2801, San Miguel, Santiago 8910060, Chile; 2 Departamento de Ciencias Animales, Facultad de Agronomía e Ingeniería Forestal, Pontificia Universidad Católica de Chile, Macul, Santiago 7820436, Chile; romina.abarca@uc.cl; 3 Instituto de Ciencias Naturales, Universidad de Las Américas, Manuel Montt 948, Providencia, Santiago 7500975, Chile; cris.linares.flores@gmail.com; 4 Departamento de Ciencias Biológicas y Químicas, Facultad de Medicina y Ciencia, Universidad San Sebastián, Campus Los Leones, Lota 2465, Providencia, Santiago 7510157, Chile

**Keywords:** chitosan, polyelectrolyte complexes, food packaging

## Abstract

The current challenges in the food packaging field are, on one side, replacing plastic from non-renewable sources with biopolymers and, on the other hand, generating a packaging material with attractive properties for the consumer. Currently, the consumer is ecologically concerned; the food packaging industry must think ahead to satisfy their needs. In this context, the utilization of polyelectrolyte complexes (PECs) in this industry presents itself as an excellent candidate for fulfilling these requirements. PECs possess enticing characteristics such as encapsulation, protection, and transportation, among others. On the other hand, diverse types of biopolymers have been used in the formation of PECs, such as alginate, cellulose, gelatin, collagen, and so on. Hence, this paper reviews the use of PECs in food packaging where chitosan forms polyelectrolyte complexes.

## 1. Introduction

At present, the planet is in a critical position because industries generate thousands of tons of waste. The marine industry is no stranger to this issue since this type of manufacturing generates waste which could produce diseases, adding to the bad smell caused. Due to the pandemic caused by COVID-19, the production of food and marine products decreased because of the lockdown, which caused precariousness in the food supply; however, this had a slight increase in 2021 [1].

According to FAO (Food and Agriculture Organization) data, world production of crustaceans reached around 11 million tons in 2020; the above implies that the amount of waste generated is important [1]. Currently, the recycling of waste is a common necessity to diminish environmental damage. In this way, the use of waste from crustaceans is used for obtaining chitin and then chitosan. Chitin is a polysaccharide that is a major component present in the hard outer shells of shrimp and lobsters. From the alkaline hydrolysis of chitin, chitosan can be obtained [2]. Chitosan is a recognized linear polysaccharide biopolymer with amine and hydroxyl groups in its chemical structure, providing unique features that modify it chemically [3,4,5]. In addition, chitosan possesses antimicrobial characteristics, which turn into a biopolymer attractive for applications in various fields, from the pharmaceutical to the food industry [6,7,8,9].

On the other hand, over the past decade, the application of nanotechnology from environmentally friendly materials has emerged as a new field that utilizes nanoscale materials to deliver drugs, genes, and imaging agents. Thus, a wide variety of studies regarding nanoaggregates in several applications have been published [10,11,12].

**Figure 1:**
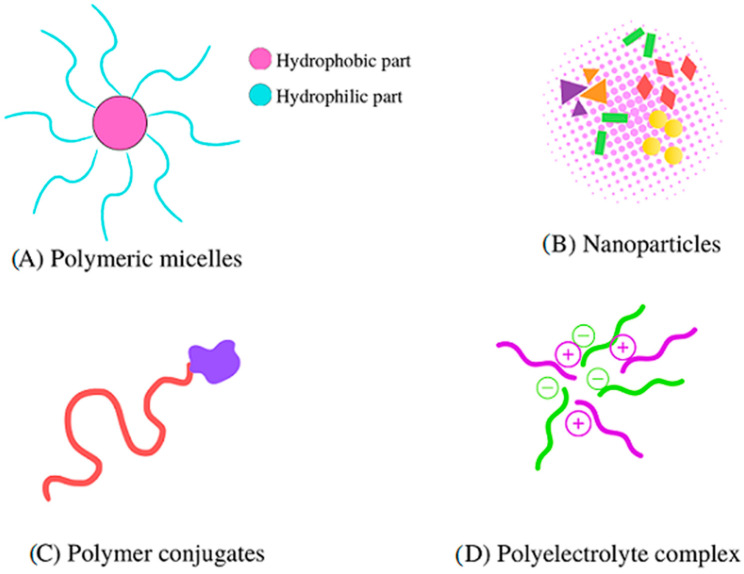
Scheme of different types of nanoaggregates systems.

Among the different types of nanoaggregates systems that have been elaborated can be identified: polymeric micelles, nanoparticles, polymer conjugates, and polyelectrolyte complexes (PECs) (Figure 1). 

**Figure 2:**
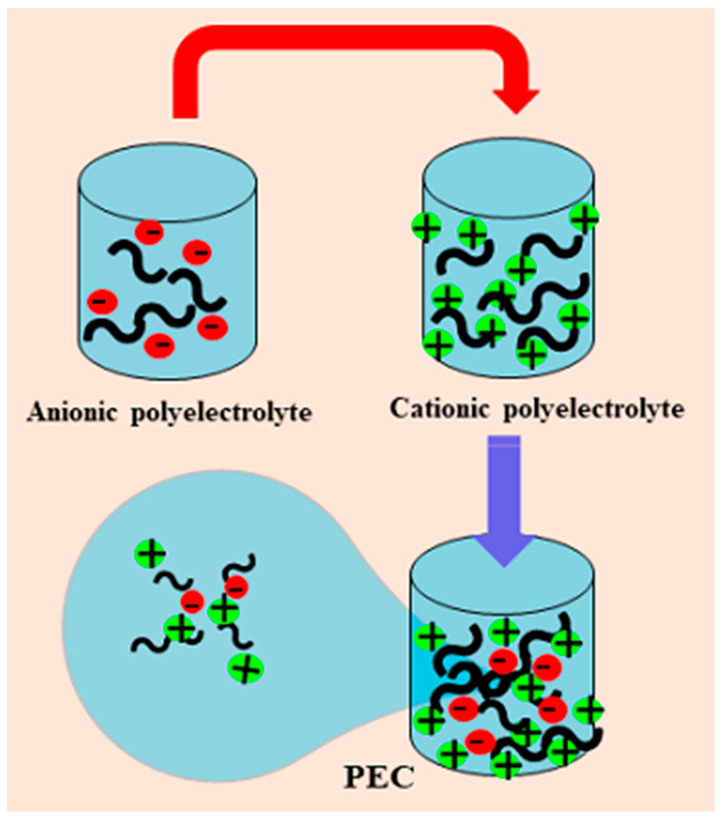
Scheme of formation of PEC system.

These systems are mainly used as carriers or reservoirs of bioactive molecules. Among the polymeric systems, PECs stand out, which can generate two environments in one structure: hydrophilic and hydrophobic. PECs are generated via long-range electrostatic interactions through charged polymers, known as polyelectrolytes (PEs). These PEs are formed from repeating units which carry charge when dissolved in water (most polyelectrolytes have high solubility in water), i.e., they generate polycations or polyanions through counterion release. Thermodynamically, it is well known that the release of the counterion is the driving force in PECs’ generation [13]. These interactions between the PEs lead to the formation of voids in the PECs formed; thereby, the PECs are capable of generating encapsulating systems, which could improve the bioavailability and the distribution of active compounds. Thus, one of the significant applications of these systems is as a carrier for delivery systems (Figure 2).

PECs are made up of charged polymers with negative and positive charges. The interaction between these two charged polymers generates stable structures known as polyelectrolyte complexes, and such structures form holes capable of accommodating small molecules [14]. 

Several factors must be considered in the formation process of PECs, such as the length of the chain of PE, charge density, stability, pH, concentration, mixing ratio, molecular weight, hydrophobicity, and ionic strength, which are some factors to take into account [15,16,17,18]. The stoichiometry of the PEs involved determines the solubility property of these systems. It is well-known that non-stoichiometric mixtures of polyelectrolytes lead to the formation of water-soluble PECs that comprise a neutral core trapping a 1:1 mixture of oppositely charged polymeric structures encircled by a shell of excess polyelectrolyte chains. In this context, knowledge of molecular weight is essential since the polyelectrolytes used must have significantly different molecular weights [19]. On the other hand, stoichiometric combinations of polyelectrolytes lead to unstable shell-deficient PECs, which flocculate due to the hydrophobic attraction between neutral coacervates [20]. In this context, solubility is managed by the stoichiometry of the PEs involved, which determines the final application of the PECs.

At the technological level, PECs are widely used because of their unique characteristics, such as water solubility and formation of voids which allow the building of systems for encapsulation, stabilization, and release of different substrates such as drugs, enzymes, antioxidants, and cells, among others. In this sense, PECs have been extensively utilized in, for example, encapsulating colorants under unfavorable conditions [21]; the development of biosensors [22]; intensification and stabilization of anthocyanins [23]; controlled release and preservation of vitamin D_3_ [24]; and transport of multivalently charged compounds [25]. In particular, they have been used as delivery systems in diverse applications including pharmaceutical and biomedical [26,27] and as a remover of metals in aqueous waste [28,29,30,31].

This review provides a contemporary look at chitosan-based polyelectrolyte complexes focused on using PECs in food packaging and shares several promising outcomes.

## 2. Polyelectrolyte Complexes Using Biopolymers 

The use of biopolymers in several fields of knowledge has already been diversified over several years due to the relevance of generating biodegradable systems with the aim of causing the minimum impact on the environment. Therefore, the use of biopolymers in the generation of polyelectrolyte complexes is not a novelty, and various works have been published using biopolymers in this type of system [32,33,34]. In this sense, chitosan has been widely studied as part of polyelectrolyte complexes because its positive charge can be used as a polycation. In the case of polyanions from biopolymers for application in PEC systems, alginate, pectin, hyaluronic acid, gum arabic, and gellan gum are some of the ones that have been used for this purpose [35,36,37,38,39,40,41,42]. 

The application of nanotechnology in different areas is well known. One of those applications is related to transporting substances such as drugs, genes, and imaging agents. Thus, a wide variety of studies regarding nanoaggregates in several applications have been published [10,12,43,44]. Currently, the use of different types of nanoaggregates is being developed. As previously noted, PEC systems are a type of nanostructure highly attractive due to their capacity for carrying active non-soluble compounds in aqueous medium compounds.

**Figure 3:**
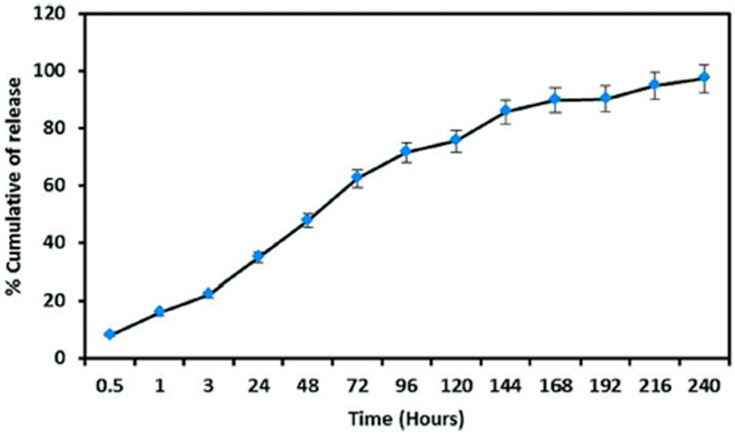
In vitro drug release profiles of umbelliprenin from nanocarriers coated with chitosan and folic acid in PBS solution. (Reproduced with permission from Ref. [45]. Copyright 2023 Elsevier).

Sadeghzadeh et al. [45] developed nanocarriers coated with chitosan and folic acid to deliver umbelliprenin. This substance is used for its highlighted cytotoxicity and, in this work, was used for studying potential anti-cancer effects. The results show an excellent bonding percentage between chitosan and folic acid, while the retention and delivery of the umbelliprenin demonstrated a positive result, as seen in Figure 3.

Following these carriers, Almeida et al. [46] investigated nanostructured carriers using chitosan and chloroaluminum phthalocyanine as a photosensitizer. The goal was to functionalize the surface of nanocarriers by using chitosan to enhance the biological activity of the transporter. The outcomes were promising in the carrier’s area.

Aqueous pollution is a topic of great interest. Several possible solutions have been studied through the years. In this vein, nanotechnology has been used as a powerful tool. For instance, Freire et al. [47] studied their adsorption capacity for dye removal from an aqueous dispersion. The results showed that the nanocomposites possess an extensive surface area. The dye-adsorption capacity displayed by nanocomposites was demonstrated against anionic dyes.

**Figure 4:**
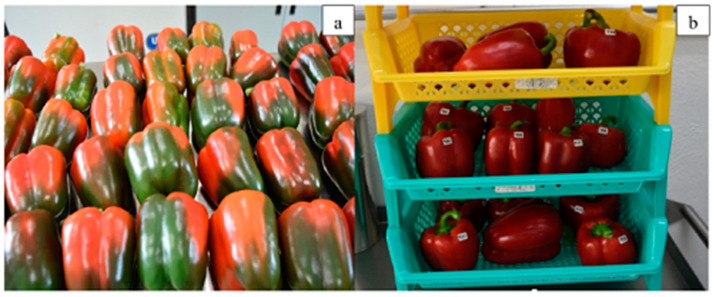
State of intermediate maturity 0 days (**a**), 21 days after different formulations with chitosan-α-pinene used (**b**). (Reproduced with permission from Ref. [48]. Copyright 2020 Elsevier).

Hernández-López et al. [48] developed a nanostructured edible coating using chitosan with α-pinene, intending to generate a material capable of preserving bell peppers’ postharvest quality and to study the coating’s resistance against the fungus Alternaria alternata. The evidence suggested that the nanostructures did not alter the flavonoids and the antioxidant capacity of bell peppers. In general, the results obtained showed that the nanostructured edible coating presented good results to be potentially applied to bell peppers (Figure 4).

Nanostructures created with graphene, multiwalled carbon, and chitosan were created via self-assembly to obtain an electrochemical sensor for sensitive detection of bisphenol A in milk samples. The systems thus created showed distinctive characteristics in electrocatalytic activity and conductivity. The combined effect of graphene, multiwalled carbon, and chitosan was responsible for this new electrochemical sensor showing good stability, repeatability, and reproducibility. The results showed that this sensor has the potential to be used for BPA identification in dairy samples with excellent accuracy and precision parameters [49]. 

**Figure 5:**
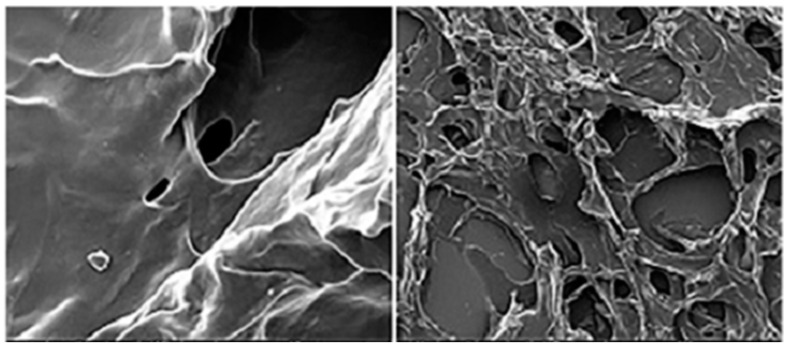
SEM image of pectin–chitosan conjugate. (Reproduced with permission from Ref. [50]. Copyright 2020 Elsevier).

In a different work, Tian et al. [50] developed a PEC system using pectin and chitosan to obtain a carrier for a delivery system; Figure 5 shows an SEM image of this formulation. The results showed that the system displays excellent properties such as mechanical strength, stability, and biodegradability. 

**Figure 6:**
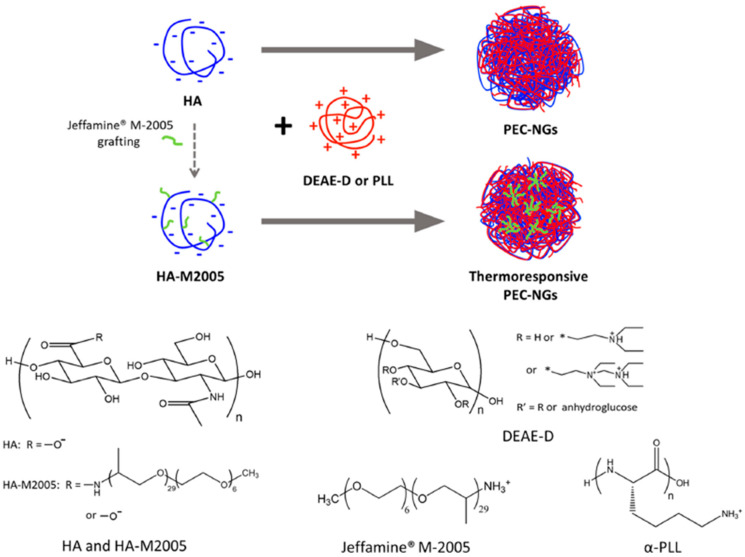
Scheme of PEC formation. (Reproduced with permission from Ref. [51]. Copyright 2022 Elsevier).

Nanogels based on polyelectrolyte complexes have been developed by Le et al. (2022). This material is a thermoresponsive one. The researchers combined functionalized hyaluronic acid with diethyl aminoethyl dextran or poly-L-lysine from the above. Depending on the mixture, different hydrophobicity grades were obtained in the PECs. Figure 6 shows a scheme of the PEC thus developed. The encapsulation of curcumin as a drug model was used. The research indicated that the systems displayed high thermoresponsiveness, stability, and encapsulation. With these qualities, the system improved the solubility of curcumin in an aqueous medium.

Table 1 shows some applications using PECs based on biopolymers as encapsulating systems.

As highlighted above, the utilization of biopolymers in polyelectrolyte complexes (PECs) presents a promising pathway for producing environmentally friendly materials. Among these biopolymers, chitosan stands out due to its numerous advantages over others. First and foremost, chitosan exhibits remarkable abundance and sustainability as it can be sourced from waste materials such as crustacean shells, as well as from renewable resources such as fungi. Additionally, chitosan demonstrates high biocompatibility and biodegradability, making it well-suited to a wide range of biomedical applications. Its low toxicity ensures minimal adverse effects, further enhancing its appeal in various fields, including biomedicine. The versatility of chitosan is evident from its ability to undergo structural modifications, facilitating the development of tailored PECs with specific functionalities. When its primary amine groups are chemically modified, chitosan can accommodate the incorporation of desired elements such as drugs and targeting ligands. This adaptability positions chitosan as a versatile platform for the creation of multifunctional PECs.

Furthermore, chitosan possesses inherent antimicrobial properties, making it an attractive material for applications requiring antimicrobial protection. This feature adds to its overall appeal in areas where safeguarding against microbial contamination is crucial.

In short, chitosan stands out from other biopolymers in the realm of polyelectrolyte complex formation, primarily due to its abundance, sustainability, biocompatibility, biodegradability, structural versatility, and antimicrobial characteristics. Its exceptional attributes make chitosan an excellent candidate for integration into PEC systems. 

## 3. Active Food Packaging

Today’s consumer is more demanding regarding the quality of packaged food, leading to the food packaging industry’s generation of packaging with more characteristics such as “smart” antimicrobial packaging for food preservation. In this sense, a large number of works have reported on the subject. Incorporating different types of structures into a matrix has various purposes, named some: increasing the resistance, porosity, and flexibility of the material [58,59,60,61,62]. The enhancement of the shelf life of the food is of great importance to the consumer. In this way, diverse structures have been introduced with antimicrobial properties, such as nanoparticles, natural products, and essential oils [63,64,65,66,67].

**Figure 7:**
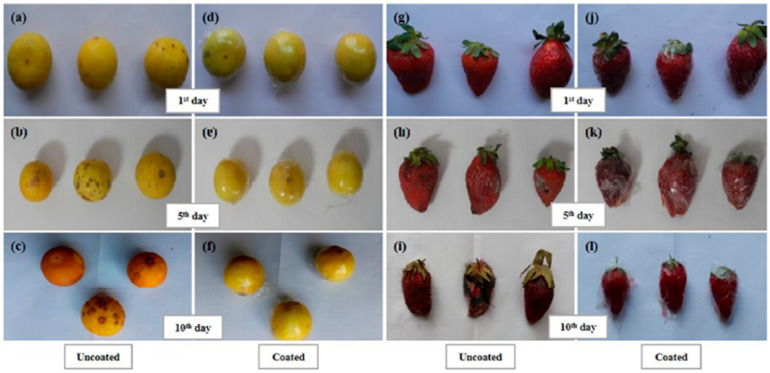
Preservation effect of AgPVA (Ag nanoparticles and poly(vinyl alcohol)) nanofibers on fruits. (**a**–**f**) uncoated and coated lemons, and (**g**–**l**) uncoated and coated strawberries. (Reproduced with permission from Ref. [68]. Copyright 2019 Elsevier).

The remarkable characteristics of nanoparticles, such as size, and antimicrobial properties, for example, are desirable for the food packaging industry. Kowsalya et al. [68] developed Ag nanoparticles and poly(vinyl alcohol) to form nanofibers to be used in films for food packaging. The system exhibited a high antimicrobial capacity against several bacteria when applied as fruit packaging. With these results, the potential shelf life of the product could be increased, as can be observed in Figure 7.

Halloysites are tubular clay nanoparticles widely studied to form part of different systems. Thanks to the tubular shape of halloysites, they can be used as an encapsulating agent. Alkan Tas et al. [69] developed a film of polyethylene coating with these tubular nanoparticles and incorporated carvacrol, which has antimicrobial characteristics, intending to improve the final material regarding the quality and security of the food. The outcomes of this investigation revealed that the halloysites incorporated in the film allowed the release of carvacrol over time. These results could enhance the quality of food and increase its shelf life.

**Figure 8:**
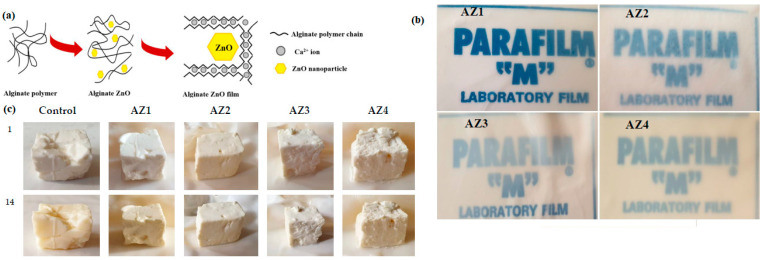
(**a**) Schematic representation of ZnO-alginate films; (**b**) transparency of the films in the different formulations studied (AZ: ZnO-Alginate-Citronella); (**c**) visual appearance of soft cheese portions packaged in alginate control film and AZ1–AZ4 films, respectively. (Reproduced with permission from Ref. [70]. Copyright 2021 MDPI).

The incorporation of more than one active compound has been studied as well. In this work by Motelica et al. [70], ZnO nanoparticles were incorporated into alginate films (Figure 8a). The authors incorporated citronella essential oil into the above formulation to observe whether the combination of these compounds generated a synergic effect. This study aimed to obtain an active biofilm with improved characteristics. The results showed improved water barrier properties; meanwhile, antibacterial activity against *B. cereus* showed promising results. The active films were tested on soft cheese, demonstrating that the shelf life was extended by over 14 days. Figure 8b,c shows the films that were obtained and films tested on soft cheese, respectively. 

Food packaging based on biodegradable materials is a subject of interest to decrease the waste from non-biodegradable materials. In their study, Abarca et al. [71] incorporated nisin and EDTA into a gelatin matrix to obtain a biodegradable film with antimicrobial properties. The results displayed an important antimicrobial effect against *Escherichia coli*. In another study of chitosan-gelatin and pectin-chitosan, films and coatings were made. The authors incorporated lemongrass essential oil, Zn, or ZnO as active compounds into the films. The thermal results showed high stability. Regarding mechanical properties, the films made from chitosan-gelatin showed good characteristics for practical applications.

Moreover, the antibacterial effect was tested, and the results showed a synergic effect between the active compounds incorporated into the films. The novelty of this study is that they tested the coating on boxes containing raspberries. The best microbiological behavior was found in boxes coated in chitosan-gelatin emulsion with ZnO. The shelf life of the fruit was prolonged by all formulations studied from four to eight days [64]. 

**Figure 9:**
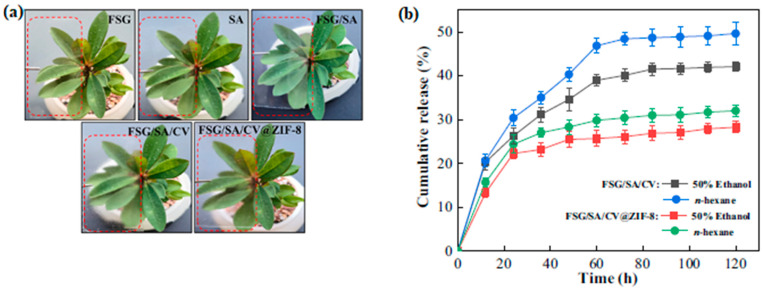
(**a**) Photographs of pure and different composite films; (**b**) cumulative release of carvacrol from different composite films into different food simulants (50% ethanol and n-hexane). (Reproduced with permission from Ref. [72]. Copyright 2023 Elsevier).

Zn nanoparticles loaded with carvacrol were developed to be incorporated into films made with fish scale-derived gelatin and sodium alginate (Figure 9a). The mechanical properties of the film were improved, showing a good ability to stretch without breaking. Additionally, the solubility of the film in water decreased; meanwhile, the thermal stability improved as well. Regarding the antibacterial activity of the films, the findings indicated that a good response was noted against *E. coli* and *S. aureus*, and the carvacrol release from the films in different food simulants showed excellent results, as can be observed in Figure 9b. The researchers believe this film might be used as food packaging for strawberries to maintain postharvest quality [72].

**Figure 10:**
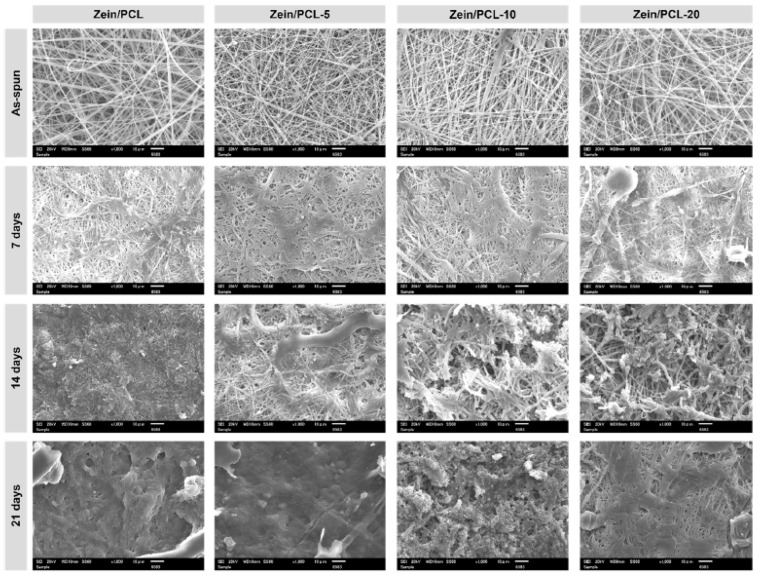
SEM images of the neat and BC-HNT (β-caryophyllene-halloysite nanotubes)-loaded Zein/PCL (polycaprolactone) nanofibers as a function of composting time. (Reproduced with permission from Ref. [73]. Copyright 2023 Elsevier).

Various kinds of active films have been developed across the years to improve specific poor characteristics of biofilms, such as elasticity, barrier properties, and toughness. Over time, many substances have been created with these goals. To this end, Ullah et al. [73] used zein protein together with polycaprolactone in electrospun nanofiber sheets into which halloysite nanotubes (HNT) were incorporated. In addition, they incorporated β-caryophyllene, which is a bicyclic sesquiterpene with high anti-inflammatory, antibacterial, and antioxidant properties, among others. Figure 10 shows SEM images of the nanofibers studied. This work showed that the mechanical and thermal characteristics of nanofiber sheets were enhanced by adding halloysite. The material was tested on strawberries in storage conditions, and the results were encouraging since the encroachment of moisture was observed to be delayed. These results show that the material generated can fortify the film.

Collagen is a highlighted material in food packaging. Tang et al. [74] developed collagen films modified with quinones obtained via the oxidation of phenolic acids to improve collagen performance. The results showed that collagen was successfully modified. The films with collagen modification showed improved properties, such as increased resistance to enzyme degradation; the mechanical and thermal properties were improved as well. The antioxidant capacity of the modified material was highlighted. Meanwhile, the antimicrobial capacity against *E. coli* and *S. aureus* showed promising findings in modified films.

Ali et al. [75] developed films based on gum arabic crosslinked with butyl acrylate and hydroxyethyl methacrylate. These researchers aimed to obtain self-sticking films loaded with cinnamon essential oil to be potentially used as active packaging in the vapor phase. The encapsulation and release results of cinnamon oil showed a good performance. The antimicrobial activity assessment showed relevant results against *E. coli* inoculated in string cheese.

### Food Packaging Using Polyelectrolyte Complexes Systems Chitosan Based 

The current pace of life has led the industry to improve its products. The food packaging industry is no stranger to that. The current consumer demands that food has a long shelf life, and the industry has adjusted to this requirement. In this sense, films based on PECs offer multiple opportunities to obtain a material with remarkable properties.

Food packaging generates a large amount of waste because a large part consists of non-biodegradable materials, causing significant environmental damage. Great efforts are being made to diminish this kind of remains. Using biodegradable polymers as potential replacements in food packaging materials is one alternative for this severe situation. In this matter, chitosan, PLA (polylactic acid), and PHB (polyhydroxybutyrate) are the most biodegradable polymers studied. Among them, chitosan has been widely investigated in the most diverse areas because of some of its prominent properties, such as low toxicity, biocompatibility, cost-effective production, and high availability; it makes an excellent alternative to form part of multiple materials [76,77,78,79,80,81,82,83]. 

In this sense, the studies on using chitosan in the food packaging industry have been widely covered. The antimicrobial properties of chitosan have been a great advantage when choosing what material to use because of the possibility of extending the shelf life of packaged food, as was reviewed in the previous section.

The attractive properties of PECs in encapsulation make them highly desired substances since they have unique properties. Because of that, PEC films in food packaging have been studied as well. Kurek et al. [84] developed a film based on PEC with chitosan and pectin as PEs. Into the PECs, they put blackcurrant powder (from blackcurrant waste) as a pH indicator and for its antioxidant properties. This study showed that blackcurrant was successfully incorporated into PECs, and changes in color were linked to different pH values, from acid to alkaline, showing high effectivity to be used as smart food packaging.

The use of PECs in food packaging points to creating smart materials. To this end, Şen et al. [85] and Torres Vargas et al. [86] studied a film based on PECs using alginate and yucca starch with the incorporation of extracts of natural origin (anthocyanin and betanin from the exocarp of black eggplants and the mesocarp of beets) as an indicator. The goal was to generate a smart material. The outcomes indicated that the films prepared effectively have a sensor property because of the addition of natural extracts. Good general properties of films based on PECs were obtained for this group—good thermal, surface, and antioxidant properties.

**Figure 11:**
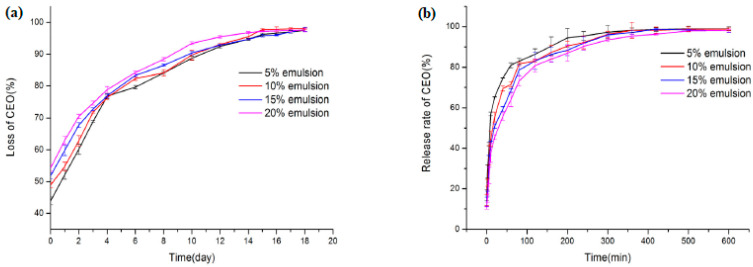
The growth-inhibiting activity of chitosan-based films on *E. coli* (**a**) and *S. aureus* (**b**). (Reproduced with permission from Ref. [87]. Copyright 2020 Elsevier).

Cinnamon essential oil emulsions were stabilized by gum arabic modified with octenyl succinic anhydride. Gum arabic is an anionic polyelectrolyte, while chitosan is a cationic polyelectrolyte. Thus, in this work, the authors prepared chitosan-based polyelectrolyte films. The active substance, cinnamon essential oil, was highly retained in the films—this enhanced antimicrobial activity against *E. coli* and *S. aureus* [87]. Figure 11 shows the growth-inhibiting activity of the different concentrations used.

The water vapor barrier is an essential characteristic in films for food packaging applications. Unfortunately, biopolymers have poor barrier properties, which can be improved through chemical modification or combination with other compounds. 

Looking to improve these properties, Chi and Catchmark [88] developed films with polyelectrolyte complex systems using crystalline nanocellulose, chitosan, and carboxymethyl cellulose. The films were successfully developed. The goal of combining these three biomaterials is mainly to enhance the characteristics of the films, such as mechanical and barrier properties.

**Figure 12:**
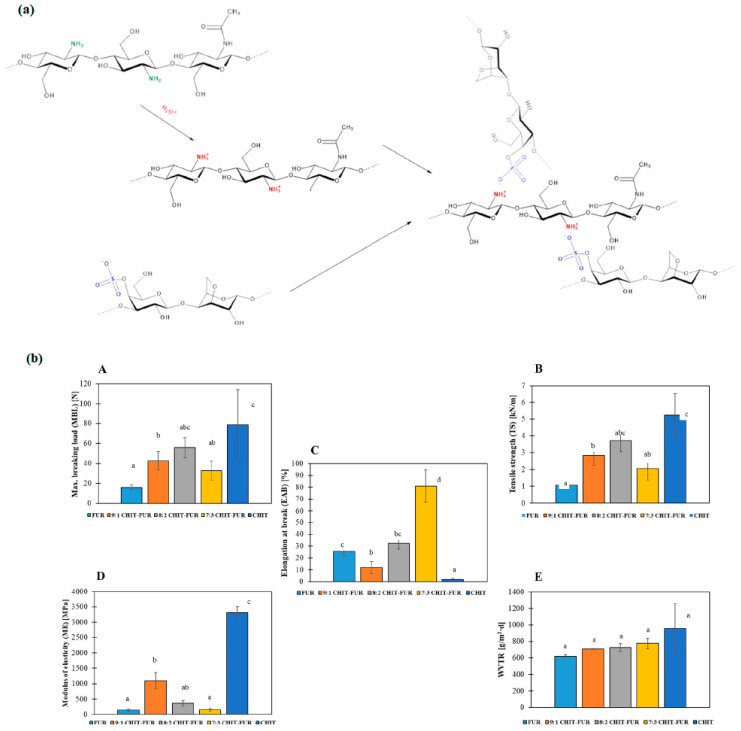
(**a**) Scheme of potential interaction between furcellaran and chitosan; (**b**) Mechanical properties ((**A**) Maximum breaking load; (**B**) tensile strength; (**C**) elongation at break; (**D**) modulus of elasticity) and (**E**) water vapor transition rate of furcelleran, chitosan films, and their complex. The same lower letters in each column demonstrate lack of significant difference between values (*p* ˂ 0.05). (Reproduced with permission from Ref. [89]. Copyright 2021 Elsevier).

Jamróz et al. [89] successfully developed films using chitosan and furcelleran. Furcelleran is an anionic polysaccharide sourced from the red algae *Furcellaria lumbricalis*. Figure 12a shows possible interactions between chitosan and furcelleran. The films obtained from the generation of PECs exhibited good thermal, mechanical, and barrier properties; the graphics of the last two properties are shown in Figure 12b. The final material could be used in food packaging applications.

The system formed with chitosan and gellan gum, both polyelectrolytes, was studied to obtain multilayer PEC films with the integration of thyme essential oil to obtain a material that could be used for food packaging [90]. The mechanical properties of the films, flexibility in particular, were enhanced thanks to the incorporation of thyme essential oil; however, the water barrier properties were decreased. The addition of thyme essential oil as an antimicrobial agent was observed, and the films showed high antimicrobial activity. This study incorporated the antimicrobial agent into the multilayer films via direct emulsion and nanoemulsion. In the latter, stronger antimicrobial activity was observed.

**Figure 13:**
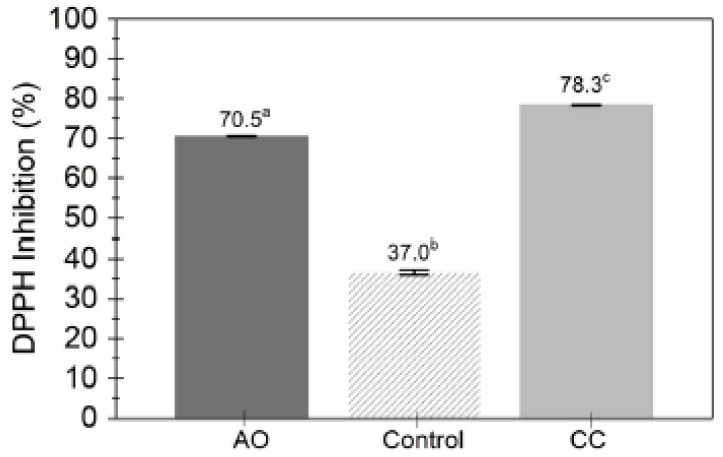
Antioxidant activity for AO (Assai pulp oil), the control and CC (Alginate-chitosan/AO) PEC. (Reproduced with permission from Ref. [53]. Copyright 2020 Elsevier).

The use of complexes, as has already been said above, is to encapsulate active compounds. To this end, Teixeira-Costa et al. [53] generated PECs with chitosan and alginate to create microcapsules. Assai pulp oil is an encapsulated compound, and its high polyphenol content is among its characteristics. The microcapsules were satisfactorily obtained, showing an excellent antioxidant capacity (Figure 13). The authors pointed out that this material could be applied to films for food packaging.

The use of cellulose for food packaging applications is vast. To this end, cationic hydroxyethyl cellulose was blended with sodium alginate. The researchers obtained uniform films with antimicrobial activity. This combination may be employed as a material for food packaging with added value since this material could increase the shelf life of food [91].

Eugenol is a phenolic derivative obtained from clove oil with broad antioxidant, antifungal, and antibacterial properties. In a study by Riyandari et al. [92], they studied the release of eugenol from polyelectrolyte complex films formed with chitosan and alginate. The outcomes showed that the release of eugenol was controlled by alginate concentration. The thermal, mechanical, water permeability and antioxidant characterization showed that those films could be applied as antioxidant material in food packaging.

**Figure 14:**
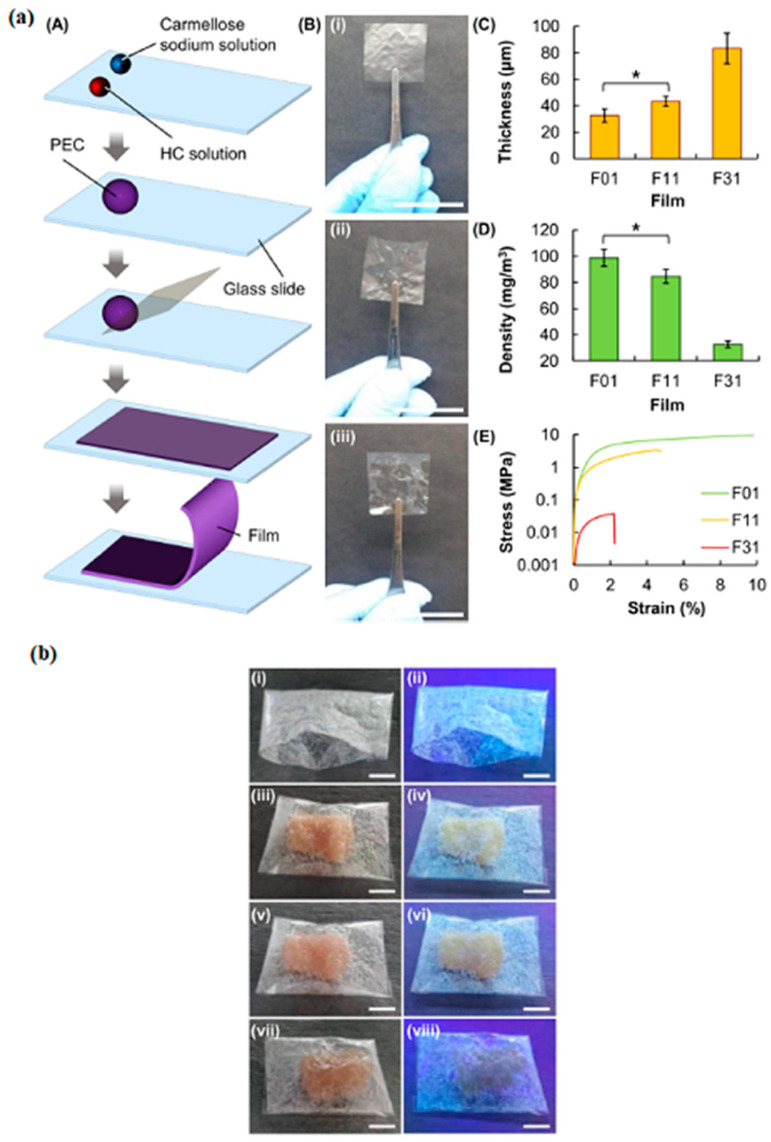
(**a**): (**A**) schematic diagram showing the procedure for film preparation. (**B**) Photos of films with different ratios of hypromellose-graft-chitosan (HC) and carmellose sodium. The thickness (* denotes *p* < 0.05) (**C**), density (* denotes *p* < 0.05) (**D**), and stress–strain curves (**E**) of different film samples. (**b**): Photos of the bag generated, as well as the bag containing (**iii**,**iv**) fresh chicken meat, (**v**,**vi**) frozen chicken meat, and (**vii**,**viii**) chicken meat thawed after being frozen, under (**i**,**iii**,**v**,**vii**) white light and (**ii**,**iv**,**vi**,**viii**) UV light. (Reproduced with permission from Ref. [93]. Copyright 2021 Elsevier).

The increase in shelf life is a subject of high interest for today’s consumers. To this end, Lai et al. [93] utilized hypromellose-graft-chitosan (hydroxypropyl methylcellulose) and carmellose sodium (sodium carboxymethyl cellulose) to form polyelectrolyte complex films. The transparent films thus generated showed good properties such as barrier, mechanical, and antibacterial activity. Figure 14a shows the films obtained and their mechanical properties as a graphic. The density of the films is shown as well. However, the most highlighted characteristic of the films was that they showed luminescent properties, allowing the consumer to see changes in the packaging in freezers, for example. Figure 14b shows the photos obtained for the films studied.

**Figure 15:**
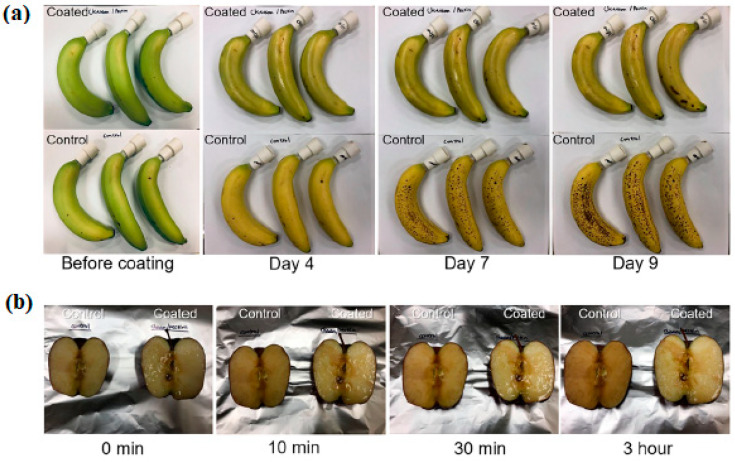
(**a**) Comparison of CH (chitosan)/PT (pectin)-coated and uncoated banana ripening as a function of time. Bananas were aged under ambient conditions. (**b**) Comparison of CH/PT-coated and uncoated apple browning as a function of time under ambient conditions. (Reproduced with permission from Ref. [94]. Copyright 2021 American Chemical Society).

Keeping food fresh is a human necessity to avoid food waste, and freshness of food is a quality critical to the consumer. In fruits and vegetables, this variable is significant. Chiang et al. [94] developed PEC-based edible coatings with chitosan and pectin to be applied to fruits. The tests carried out on fruits showed excellent barrier properties. These results are auspicious, as shown in Figure 15, since they indicate that this could be a material to be applied to fruits, increasing their shelf life.

Chitosan and alginate have been used in many studies as PEC systems. In this case, Ty et al. [95] used this system together with cinnamon essential oil as a film for food packaging meat pork in storage conditions. Keeping the meat in good condition for several days is challenging for the industry, and the researcher found that the final material presents good antioxidant and antimicrobial characteristics. The application of PEC over meat showed a reduction in microbiota, and the shelf life of raw meat was prolonged by at least twelve days.

**Figure 16:**
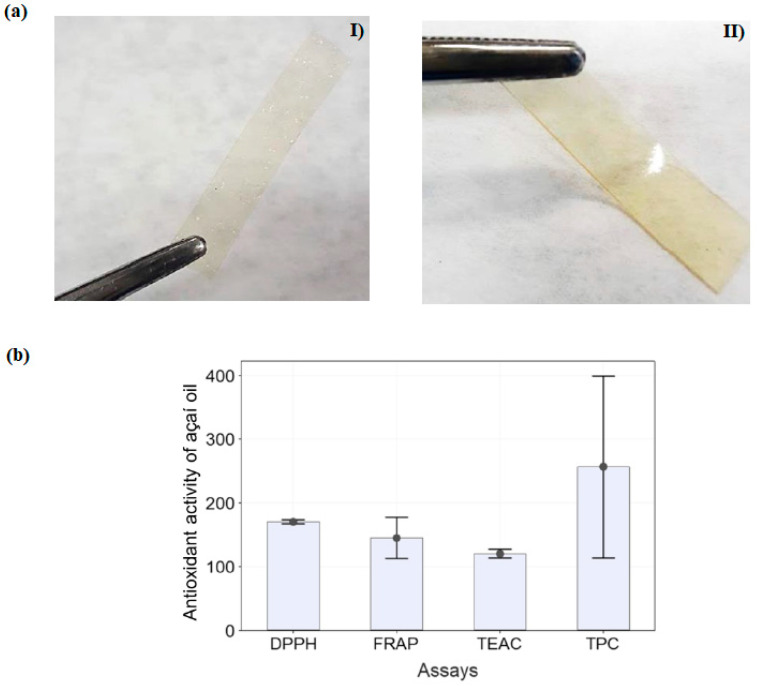
(**a**) Photographs of sample films (**I**) F0/0 (chitosan) and (**II**) F5/0 (chitosan-deep eutectic solvent) films; (**b**) antioxidant activity of açaí oil (Reproduced with permission from Ref. [96]. Copyright 2023 MDPI).

Using plasticizers on biodegradable films is crucial since these films have poor mechanical properties. Thus, deep eutectic solvents are currently a promising alternative. They are known to be sustainable, biodegradable, thermally stable, low in volatility, and non-flammable, among some attractive characteristics for plasticizers in food packaging films. Teixeira-Costa et al. [96] utilized choline chloride as a deep eutectic solvent on chitosan films. PEC microcapsules of chitosan-alginate containing açaí oil, which is recognized for its antioxidant properties, were added to these films. The homogeneous films obtained resulted in good mechanical and antioxidant properties. Figure 16a shows the films obtained, and Figure 16b shows the antioxidant activity results. The researchers observed that incorporating açaí oil microparticles influenced properties such as flexibility, thickness, and crystallinity. Regarding using a deep eutectic solvent on the films, they observed that the mechanical properties experimented with produced excellent results. The results showed that this study is an excellent alternative that could be applied to food packaging.

Tragacanth gum is a polysaccharide which is water-soluble, odorless, and tasteless and is obtained from dried sap from several species. This compound possesses good barrier properties. Chitosan and tragacanth gum, in combination, were used to create films oriented to the food packaging industry. The results showed that the films presented good mechanical properties. The shelf life of strawberries was increased using chitosan/tragacanth gum PEC films compared to polyethylene films. These results show an attractive alternative to using a PEC film to replace plastic non-biodegradable in food packaging [97].

## 4. Overview

Currently, the damage caused to the environment due to the accumulation of waste from food packaging is huge. Hence, the current challenge of this industry is the use of biodegradable materials to diminish environmental damage. At the same time, the consumer is becoming more demanding, which means that the industry must rise to the challenge. Using PEC systems as encapsulating agents in food packaging is auspicious since these systems can protect, deliver, and release the compound of interest. Antioxidants, antimicrobials, thermoresponders, and sensors are fascinating in the food packaging industry. In this review, it has been shown based on various studies that use PECs systems that using chitosan in films can produce a material attractive to the consumer. The use of chitosan in these systems turns into a very interesting product; due to the fascinating properties of chitosan, such as low cost, antimicrobial qualities, and biocompatibility, it has become a coveted biopolymer. However, there some challenges that need to be addressed, such as:

- Material stability and barrier performance: chitosan-based PEC must exhibit robust barrier properties to protect food products from external factors such as moisture, oxygen, and light. Ensuring long-term stability and maintaining the desired barrier performance of PEC films or coatings during storage and transportation is crucial. Addressing challenges related to film integrity, mechanical strength, and maintaining barrier properties over time is essential.
- Compatibility with food products: chitosan-based PECs must demonstrate compatibility with a wide range of food types, including those with varying pH levels, fat content, and water activity. Compatibility encompasses factors such as preserving taste, texture, and nutritional quality of the packaged food.
- Scalability and manufacturing efficiency: developing scalable and cost-effective manufacturing processes for chitosan-based PECs is a significant challenge. Efficient production methods are needed to meet the demands of the food packaging industry while ensuring consistent quality and performance. Optimizing chitosan extraction, purification, and film-forming techniques, as well as exploring novel processing technologies, are important areas of research.
- Regulatory compliance and safety: compliance with food contact regulations and ensuring consumer safety are critical considerations. Chitosan-based PECs must meet regulatory requirements related to migration limits, toxicity, and overall safety.
- Shelf life and preservation: maintaining the shelf life and freshness of packaged foods is essential for food quality and consumer satisfaction. These systems should effectively protect food products from microbial growth and enzymatic degradation, thereby extending product shelf life. Addressing challenges related to antimicrobial activity, control of enzymatic degradation, and maintaining sensory attributes of packaged foods is crucial.

Addressing these challenges will facilitate the successful integration of PECs chitosan-based in food packaging, offering sustainable and improved packaging options.

## Figures and Tables

**Table 1:** Some polyelectrolyte complex systems based on biopolymers and their applications as encapsulant agents. (A brief survey from 2018 to 2023 years).

Polyelectrolyte Complex System/Encapsulated Molecule	Application	Ref.
Pea-protein succinylated-Chitosan/curcumin	Delivery of curcumin in a gastrointestinal system	[52]
Chitosan-alginate/assai pulp oil	Active food packaging	[53]
Casein-sodium alginate/vanillin	Delivery systems in various areas, such as food packaging, textiles, cosmetics	[54]
Carboxymethylagarose-chitosan/diclofenac sodium	Wound dressing for transdermal drug delivery, tissue engineering	[55]
Glycosaminoglycans-chitosan/mesenchymal stem cells	Applications in bioprinting, modular tissue engineering, or regenerative medicine	[56]
Chitosan-fucoidan/platelet-rich plasma	Use in diabetic wound care	[57]

## Data Availability

Not applicable.
